# Prebiotic Properties of Non-Fructosylated α-Galactooligosaccharides from PEA (*Pisum sativum* L.) Using Infant Fecal Slurries

**DOI:** 10.3390/foods9070921

**Published:** 2020-07-13

**Authors:** María del Carmen Marín-Manzano, Oswaldo Hernandez-Hernandez, Marina Diez-Municio, Cristina Delgado-Andrade, Francisco Javier Moreno, Alfonso Clemente

**Affiliations:** 1 Estación Experimental del Zaidín (CSIC), Consejo Superior de Investigaciones Científicas (CSIC), 18008 Granada, Spain; mamen.marin.manzano@gmail.com (M.d.C.M.-M.); cristina.delgado@eez.csic.es (C.D.-A.); 2 Institute of Food Science Research (CIAL, CSIC-UAM), 28049 Madrid, Spain; o.hernandez@csic.es (O.H.-H.); m.dmunicio@gmail.com (M.D.-M.); javier.moreno@csic.es (F.J.M.)

**Keywords:** galactooligosaccharides, GOS, gut microbiota, pea, prebiotic, raffinose oligosaccharides, short-chain fatty acids (SCFA)

## Abstract

The interest for naturally-occurring oligosaccharides from plant origin having prebiotic properties is growing, with special focus being paid to supplemented products for infants. Currently, non-fructosylated α-galactooligosaccharides (α-GOS) from peas have peaked interest as a result of their prebiotic activity in adults and their mitigated side-effects on gas production from colonic bacterial fermentation. In this study, commercially available non-fructosylated α-GOS from peas and β-galactooligosaccharides (β-GOS) derived from lactose were fermented using fecal slurries from children aged 11 to 24 months old during 6 and 24 h. The modulatory effect of both GOS on different bacterial groups and bifidobacteria species was assessed; non-fructosylated α-GOS consumption was monitored throughout the fermentation process and the amounts of lactic acid and short-chain fatty acids (SCFA) generated were analyzed. Non-fructosylated α-GOS, composed mainly of manninotriose and verbascotetraose and small amounts of melibiose, were fully metabolized and presented remarkable bifidogenic activity, similar to that obtained with β-GOS. Furthermore, non-fructosylated α-GOS selectively caused an increase on the population of *Bifidobacterium longum* subsp. *longum* and *Bifidobacterium catenulatum/pseudo-catenulatum.* In conclusion, non-fructosylated α-GOS could be used as potential ingredient in infant formula supplemented with prebiotic oligosaccharides.

## 1. Introduction

The colonisation of the gastrointestinal tract (GIT) by microorganisms is an essential process in our life cycle and starts during the gestation period [1,2]. Evidence shows that the GIT colonisation rapidly increases after birth by aerobic microorganisms that decrease the concentration of oxygen. This lowering of redox potentially allows the colonisation of anaerobic bacteria [3]. The continued succession of microorganisms during the first years of life has an important effect on the long-term maturity of the GIT microbiota, which is affected by diet, mode of delivery, antibiotic treatments, genetics, intestinal mucin glycosylation and other factors [4].

Non-digestible dietary ingredients are capable of modulating composition and metabolic function of gut microbiota. In this context, the prebiotic concept was recently defined by the International Scientific Association for Probiotics and Prebiotics (ISAPP) as “a substrate that is selectively utilized by host microorganisms conferring a health benefit” [5]. It is now recognized that the prebiotic effect extends beyond bifidobacteria and lactobacilli and includes other genera such as *Akkermansia* and *Faecalibacterium*. These bacterial groups, among others, are involved in the production of lactic acid and short-chain fatty acids (SCFA), which perform crucial physiological functions as metabolic regulators and immunomodulators, decreasing the growth of potential intestinal pathogens. The SCFA are responsible for the decrease of pH in the intestinal lumen, an increase of calcium absorption and shortening of gastrointestinal transit time. Besides, SCFA play an important role in cross-feeding processes and are major sources of energy for colonic epithelial cells and other cell types located in peripheral tissues [4,6].

The human-milk oligosaccharides (HMOs) are one of the most important dietary ingredients modulating the infant microbiome. Only certain bifidobacterial species, such as *Bifidobacterium longum* subsp. *infantis*, are able to utilize HMOs as metabolic substrates [7] with specific preferences depending on the *Bifidobacterium* strain and the HMOs structure [8,9]. Due to different clinical and social conditions, human milk is not a realistic choice for some infants and, in such cases, infant formulas are the most suitable alternative. However, access to HMOs for infant formula is extremely limited [10]. As a result, different oligosaccharides have been utilised to mimic the function of HMOs, such as fructooligosaccharides (FOS) and β-galactooligosaccharides (β-GOS). Other natural sources have also been utilised to obtain carbohydrates for infant formulas with well reported prebiotic activities such as inulin [11], pectin-oligomers [6], raffinose [12] and resistant starch [13]. For infant formulas, research has mainly focused on inulin, fructooligosaccharides (FOS) and β-galactooligosaccharides (GOS); indeed, there is still a wide range of oligosaccharides that could mimic, at least to some extent, the functional properties of HMOs.

Plant-based GOS, with α-galactosidic linkages instead of β-linkages, are water soluble carbohydrates and are particularly abundant in legume seeds as soybean, chickpea, lentil, faba bean and pea. They are commonly named raffinose family oligosaccharides (RFOS) and show a terminal sucrose unit linked by the glucose monomer to galactoses via α-(1→6) linkages. Like β-GOS, α-GOS are not hydrolysed in the upper gastrointestinal tract due to the absence of the enzyme α-galactosidase expressed by somatic cells in the mammalian GIT [14]. As a result, they reach the large intestine where are fermented by gut microbiota and can exert prebiotic properties [15]. However, it is well-known that RFOS cause discomfort, bloating and flatulence to consumers due to the presence of the ending fructose monomer [16,17].

Non-fructosylated α-GOS include melibiose (CAS 585-99-9), manninotriose (CAS 13382-86-0) and verbascotetraose (CAS 1111-08-6), which are essentially raffinose, stachyose and verbascose without the ending fructose units. Non-fructosylated α-GOS from peas is produced by the activity of β-fructosidases which are able to split sucrose from the α-GOS chain into glucose and fructose, being commercially available as AlphaGOS^®^. This particular α-GOS mixture has claimed to reduce the post-prandial glycaemic responses, being recently approved by the European Food Safety Agency (EFSA) [18], and exert prebiotic activity in adult faecal inoculum [19]. Available scientific data suggest that the administration of prebiotic oligosaccharides to healthy infants does not raise safety concerns with regards to adverse effects. In this sense, non-fructosylated α-GOS has been proven safe in infant formula in a concentration up to 8 mg/mL after preclinical evaluation in neonatal piglets [20].

Despite the various scientific evidence available regarding non-fructosylated α-GOS derived from peas, no reports have been published so far regarding the effect of these plant oligosaccharides on infant microbiota either in vitro or in vivo. Taking into account the published data reporting the bifidogenic properties of non-fructosylated α-GOS in adults, and its safety in infant formula, we hypothesized that a similar effect could be also found in infants, despite the differences between GIT microbiota in adults and in infants. Such increase in the number of bifidobacteria might be considered a major shift in the gut microbiota towards a potentially healthier composition. Important functions have been attributed to bifidobacteria including a protective role against pathogens, promote gut epithelium integrity and modulate the host immune system. Therefore, the main aim of the study was to evaluate the effect of non-fructosylated α-GOS in the microbiota present in infant faecal samples with particular attention paid to their bifidogenic properties.

## 2. Material and Methods

### 2.1. Chemicals

MTBSTFA (N-terbutil-dimetil-silil-N-metil-trifluoroacetamid) was purchased by Fluka Chemie AG (Buchs, Switzerland). Short chain fatty acids (SCFA) and organic acids were obtained from Sigma-Aldrich. All other chemicals were of analytical grade.

### 2.2. Non-Fructosylated α-GOS and β-Galactooligosacharides (GOS) Characterization

Non-fructosylated α-galactooligosaccharides (α-GOS, commercial brand AlphaGOS^®^) derived from pea seeds were kindly provided by Olygose (Venette, France). The chemical composition of α-GOS is detailed in Table 1 and shows as major components mannitriose and verbascotetraose with degree of polymerization of three (DP3) and four (DP4), respectively.

An industrially available galacto-oligosaccharide mixture derived from lactose (β-GOS) was used in this study for comparative purposes as prebiotic reference. This mixture was initially composed by galactose (1%), glucose (21%), DP2 (37%), DP3 (22%), DP4 (11%), >DP4 (8%). Gel filtration chromatography was carried out for the removal of mono- and disaccharides due to the presence of high levels of digestible carbohydrates including lactose; the DP of collected fractions was determined by electrospray ionization mass spectrometry (ESI-MS). The purified β-GOS mixture consisted in a complex mixture of oligosaccharides from DP3 to DP6 and fully free of mono- and disaccharides. The trisaccharide fraction of β-GOS (35.2% of total carbohydrates) contained mainly 4′-galactosyl-lactose (15.8%), 6′-galactosyl-lactose (5.3%), as well as other minor galactobioses linked to the reducing glucose unit by β-(1→2) and β-(1→6) glycosidic linkages (Supplementary Materials Table S1) [21].

### 2.3. Infant Faecal Samples

Parents/guardians of children of the Los Angeles Nursery (Granada, Spain) were invited to attend a meeting where the nature of the study was explained and those who agreed to participate signed the informed consent. Parents/guardians were given a small survey to learn about the overall health and eating habits of donors. The participants fed on solid food and none of them had taken probiotics, fermented foods, antibiotics or received medical treatment that could affect the intestinal microbiota at least a month before the stool samples were collected. The study protocol was conducted in accordance with the ethical recommendations of the Declaration of Helsinki.

Stool samples were collected from eight children in the age range between 11–24 months. They were taken by our laboratory staff after deposition in nappies, avoiding the edges and the area in contact with the nappy and also avoiding urine that was absorbed in the cellulose. Immediately after deposition, fecal samples were housed in closed jars containing Anaerocult A tablets (Anaerocult^®^, Darmstad, Germany) in order to maintain anaerobic conditions during the transport to the laboratory. The fecal samples (~5 g), within 2 h after collection, were homogenized in a stomacher by dilution 1/10 (*w*/*v*) with anaerobic 0.1 M sodium phosphate buffer, pH 6.8, by using sterile plastic filter bags, and filtrated through Miracloth (Calbiochem, Darmstad, Germany) to remove solid residues.

### 2.4. In Vitro Faecal Fermentation

Faecal homogenates were transferred into sterile Hungate tubes containing a specific basal medium for cultivation of infant faeces (BMIF) by dilution 1/10 (*v*/*v*) [22] and were stabilized overnight under anaerobic conditions at 37 °C. After that, 0.3% (*w*/*v*) of specific substrate (non-fructosylated α-GOS and β-GOS) was added to stabilized faecal mixtures. Fermentation experiment for the fecal sample of each subject was carried out once for each GOS-type. An additional tube was kept without inoculum and without GOS as a negative control and the positive control contained inoculum but not GOS. In addition, the medium was supplemented with 1/1000 (*w*:*v*) of resazurine to control the strict anaerobiosis conditions. Fermentations were carried out in anaerobic chambers at 37 °C and samples were taken at time 0, 6 and 24 h. One mL of culture was centrifuged (12,000× *g* for 15 min). The pellet was frozen at −80 °C and freeze-dried for the study of bacterial populations; the supernatant was used for direct determination of pH at the different collection times (Crison Instruments S.A., Barcelona, Spain) and in order to quantify lactic acid and SCFA.

### 2.5. DNA Extraction

DNA was extracted from pellets of fecal batch cultures using FavorPrep™ Stool DNA Isolation Mini Kit (Favorgene Biotech Corp, Shuttleworthstraße, Vienna, Austria). A NanoDrop ND-100 spectrophotometer (NanoDrop Technologies, Wilmington, DE, USA) was used for DNA quantification; purified DNA samples were stored at −80 °C.

### 2.6. Microbial Faecal Populations Determined by Quantitative PCR (qPCR) Analysis

Quantitative PCR was used to evaluate the effect of non fructosylated α-GOS and β-GOS on microbial composition in fecal homogenates after 6 and 24 h of in vitro fermentation in comparison with the control group (absence of oligosaccharides). Different microbial groups including total bacteria, Bacteroides, lactobacilli, bifidobacteria, *Eubacterium rectale*/*Clostridium coccoides*, *Clostridium leptum*, enterobacteria and *Faecalibacterium prausnitzii* were distinguished and quantified using qPCR. The 16S rRNA gene-targeted group-specific primers used in this study are listed in Supplementary Materials Table S2. qPCR conditions used in this study were as previously reported [23]. For quantitative analysis of the different bifidobacteria species (*B. adolescentis*, *B. bifidum*, *B. catenulatum*/*pseudo-catenulatum*, *B. breve*, *B. longum* subsp. *infantis* and *B. longum* subsp. *longum*), the PCR conditions were one cycle of 94 °C for 5 min, then 40 cycles of 94 °C for 20 s, 55 °C for 20 s, and 72 °C for 1 min. The species-specific primer pair for bifidobacteria is listed in Supplementary Materials Table S3. In the case of *Eubacterium rectale*/*Clostridium coccoides* and *Clostridium leptum* groups, PCR conditions were an initial denaturation step at 94 °C for 5 min followed by 40 cycles at 94 °C for 20 s, 50 °C for 20 s and 72 °C for 1 min for primer annealing and product elongation [23]. The fluorescent product was detected in the last step of each cycle. Following amplification, melting temperature analysis of PCR products was performed to determine the specificity of the PCR. The melting curves were obtained by slow heating at 0.5 °C increments from 55 to 95 °C, with continuous fluorescence collection. A plasmid standard containing the target region was generated for each specific primer set using DNA extracted from fecal homogenates. The amplified products were cloned using the TOPO TA cloning kit for Sequencing (Invitrogen, Barcelona, Spain) and transformed into *E. coli* One Shot Top 10 cells (Invitrogen). Sequences were submitted to the ribosomal RNA database to confirm the specificity of the primers. For quantification of target DNA copy number, standard curves were generated using serial 10-fold dilutions of the extracted products by using at least six non-zero standard concentrations per assay. The bacterial concentration in each sample was measured as log_10_ copy number by the interpolation of the *C_t_* values obtained by the fecal homogenates samples and the standard calibration curves. Each plate included triplicate reactions per DNA sample and the appropriate set of standards.

### 2.7. Short-Chain Fatty Acids (SCFA) and Lactic Acid Analysis

SCFA and lactic acid were analyzed as described previously by Tabasco et al. [24], with some modifications. For quantitative analysis, 55 μL of a 100 mM 2-ethylbutyric acid in distilled water was added as internal standard to 550 μL of bacterial supernatant after in vitro fermentation. SCFA and organic acids were extracted by the addition of 275 μL concentrated HCl and 1 mL diethyl ether followed by vortexing 1 min. Then, samples were centrifuged at 3000× *g* for 10 min and 50 μL of the ether layer was transferred to a GC-microvial to minimize ether evaporation, adding 10 μL of the derivatization agent MTBSTFA. Hermetically stoppered microvials were heated at 80 °C for 20 min, afterwards the reaction mixture was kept for 24 or 48 h at room temperature to ensure total derivatization. An external calibration curve was built using a standard solution mixture made from pure compounds (formate 10 mM, acetate 60 mM, propionate 20 mM, butyrate 20 mM, iso-butyrate 5 mM, valerate 5 mM, iso-valerate 5 mM, lactate 17 mM, succinate 17 mM) and derivatized as described for samples. A gas chromatograph Thermo Scientific (Trace GC Ultra, Rodena, Italy) equipment was used, with a mass detector ion trap (Thermo Scientific ITQ 900, Rodena, Italy) and with an automatic sampling system (Triplus). The chromatograph was equipped with a Thermo Scientific column (TR-5MS 30 m × 0.25 mm × 0.25 μm) and a 2.5 m × 1 μm 0.32 ID pre-column (Teknokroma, Guard Column TR30005, San Cugat del Vallés, Spain). The samples were introduced via split (ratio 50:1) injection with the port heated to 275 °C. Helium was used as the carrier gas at a flow rate of 1.0 mL/min. The oven temperature was initially held at 63 °C for 3 min, increased to 190 °C with a 20 °C per min rate, held at 190 °C for 6 min, then raised to 230 °C with a 40 °C per min rate, where it was held for 1 min. The mass spectrometer interface temperature was set to 250 °C. For monitoring and confirmation analysis, the electronic impact (EI, 70 eV) mode was used. For the MS/MS experiments, the adequate ion precursor and a collision energy was selected for each compound, in order to perform the quantification. Identification and quantification of SCFA and organic acids was based on the use of the relative response factors calculated for the target compounds in the standard solutions at different concentrations against the IS. Measurements were conducted at least twice.

### 2.8. PCR-Denaturing Gradient Gel Electrophoresis (DGGE) Analysis of Bifidobacteria

The 16S rRNA genes were amplified by PCR from extracted DNA of pellets from fecal batch cultures of infants. The *Bifidobacterium* genus-specific primers (Bif164-F and Bif662-GC-R) and PCR amplification conditions were as previously reported [23]. PCR fragments were separated by DGGE by using a denaturing gradient of 40 to 65%. The gels were visualized by silver-staining, dried at 37 °C and scanned. The total number and dendogram of similarity cluster analysis of DGGE bands were determined by Quantity One analysis software (BioRad). DGGE profiles were determined by cluster analysis using the Dice similarity coefficient and the unweighted-pair group method by means of arithmetic average clustering algorithm (UPGMA). The richness (S) of the bifidobacterial community was established from the number of electrophoretic bands in individual samples. Shannon index (H), was calculated as reported by Magurran [25] as: H = −∑ (pi · lnpi), where *pi* is the abundance of every species. The evenness (E) of the bacterial community was further estimated as E = H/lnS [26].

### 2.9. Excision and Sequencing of DGGE Bands

The DGGE bands were excised and eluted by using sterilised distilled water at 4 °C. The primers Bif164-F and Bif662-R were used to amplify the bands of interest as previously described [23]. PCR products were purified and then cloned using the TOPO TA Cloning kit for Sequencing (Invitrogen). Plasmids DNA were isolated from selected transformants with the GenElute Plasmid Miniprep kit (Sigma-Aldrich, St. Louis, USA), being inserts sequenced. Searches for sequence similarity were carried out using the BLAST algorithm [27] of the GenBank database Release 232.0 (www.ncbi.nlm.nih.gov) for identification of the nearest relatives of the partial 16S rRNA sequences. A sequence similarity ≥98% of the 16S rRNA gene was used as the criterion for identification of bifidobacterial species.

### 2.10. Carbohydrate Quantification by Gas Chromatography Coupled to Flame-Ionization Detector (GC-FID)

The carbohydrate concentration before and after in vitro fecal fermentation was analysed by GC-FID. The carbohydrate fraction was derivatized to their corresponding trimethylsilyl oximes (TMSO) following the method of Brobst and Lott [28]. The samples were dried and the oximes were formed by adding 350 μL of hydroxylamine chloride in pyridine (2.5% *w*/*v*) at 70 °C for 30 min. The resulting oximes were silylated with hexamethyldisilazane (350 μL) and trifluoroacetic acid (35 μL) at 50 °C for 30 min. The mixtures were centrifuged at 7000× *g* for 4 min. The supernatants were stored at a temperature of 4 °C prior to analysis. The chromatography separation was carried out in an Agilent Technologies gas chromatograph (Mod 7890A, Santa Clara, USA) with a fused silica capillary column DB-5HT (5%-phenyl-methylpolysiloxane; 30 m × 0.25 mm × 0.10 µm) (Agilent). The oven temperature was set to 150 °C and then increased to 380 °C at a rate of 3 °C/min. The injector and detector temperatures were set to 280 °C and 385 °C, respectively. One mL/min of nitrogen was used as carrier gas and the injections were performed in split mode (1:20). Data acquisition and integration were performed using the Agilent ChemStation software.

### 2.11. Statistical Analysis

The effect of non-fructosylated α-GOS and β-GOS on microbiota composition and SCFA amounts of fecal contents was analyzed using a linear mixed model: repeat measure (SPSS Statistics version 22.0, Madrid, Spain). The Bonferroni method with a *p* value ≤ 0.05 was used for adjustments for major effects and the time 0 h was used like a covariate.

## 3. Results and Discussion

### 3.1. Microbiota Composition after In Vitro Fermentation of Non-Fructosylated α-GOS and β-GOS with Infant Fecal Samples

Using infant fecal slurries, all bacteria and up to seven different bacterial groups were analysed before and after 6 and 24 h of the in vitro fermentation of non-fructosylated α-GOS and β-GOS. Table 2 shows the population of these bacterial groups, which is the average of data resulting from eight infant donors. During treatment, the control samples (in the absence of oligosaccharides) did not show significant differences, with the exception of bifidobacteria and enterobacteria that slightly increased after 6 and 24 h. *Bifidobacterium* spp. increased significantly in non-fructosylated α-GOS and β-GOS groups but no differences were observed between 6 and 24 h treatment. Indeed, the bifidogenic effect in non-fructosylated α-GOS and β-GOS groups was significantly greater than that found in control samples. To the best of our knowledge, no data has been previously reported regarding the effect of non-fructosylated α-GOS in the modulation of infants’ microbiota, using either in vivo or in vitro systems. On the contrary, the bifidogenic properties of β-GOS in infants’ microbiota is well documented. Thus, the bifidogenic activity of β-GOS using infant fecal slurries in a three-stage in vitro culture method that mimics the proximal, transversal and distal colon has been reported [29]. A strong bifidogenic activity in an in vivo intervention study in infants by using a formula-fed containing β-GOS was also reported [30]. A significant decrease in enterobacteria was observed in non-fructosylated α-GOS and β-GOS samples, while a slight increase was found in the control group. *C. coccoides/E. rectale* group, Lactobacillus spp., *C. leptum* group, *F. praustnizii, Bacteroides* and total bacteria did not show significant differences in both non fructosylated α-GOS and β-GOS treatments (Table 2).

Analysis of different bacterial groups after in vitro fermentation of non-fructosylated α-GOS and β-GOS suggest that these oligosaccharides are preferentially metabolized by bifidobacteria. Although this study is mainly focused in the bifidogenic properties of both GOS types, prebiotic targets extend beyond stimulation of bifidobacterial and lactobacilli, and recognizes that health benefits can derive from effects on other beneficial taxa. Such is the case of *F. praustnizzii* and the *Clostridium coccoides*/*Eubacterium rectale* bacterial group that includes species that are known butyrate-producers, thereby contributing to important processes linked to colonic health, including the protection against inflammatory bowel diseases. As reported in Table 2, in vitro fermentation of non-fructosylated α-GOS and β-GOS did not affect the growth of these butyrate-producers whilst enterobacteria numbers significantly decreased. In this sense, further studies are planned to determine the preventive role of non-fructosylated α-GOS in the colonization of pathogenic enterobacteria.

The modulation of gut microbiota by RFOS has been previously evaluated using in vitro fermentation batch cultures, inoculated with a standardised human adult faecal sample; a higher increase of *Bifidobacterium* spp. using RFOS when compared with FOS and β-GOS was observed, being the bifidogenic effect dose-dependent [19]. However, to the best of our knowledge, there is not specific information regarding the potential prebiotic properties of non-fructosylated α-GOS. Recently, the prebiotic potential of melibiose-derived gluco-oligosaccharides linked by α-(1→3) and α-(1→6) glycosidic bonds as unique carbon source has been evaluated by testing the growth of bifidobacteria (*B. breve*, *B. longum* subsp. *longum*, *B. animalis*) and lactobacilli (*L. reutieri*, *L. plantarum* and *L. rhamnosus*) [31]. In agreement with our data, lactobacilli were unable to metabolize melibiose-derived oligosaccharides whereas bifidobacteria were able to utilize them.

Although no data regarding the modulatory effect of non-fructosylated α–GOS in infant fecal microbiota has been previously reported, it has been suggested they could be safely used in infant formulas. In a study carried out with neonatal piglets to mimic infancy conditions, an intervention during three weeks having a daily consumption of 3.6–3.9 g non-fructosylated GOS/kg body weight demonstrated to be well tolerated with no adverse effects in terms of clinical signs, body weight, feed consumption, haematology, organ weight and histopathology [20].

### 3.2. Effect of Non-Fructosylated α-GOS on Bifidobacterial Composition

**Figure 1:**
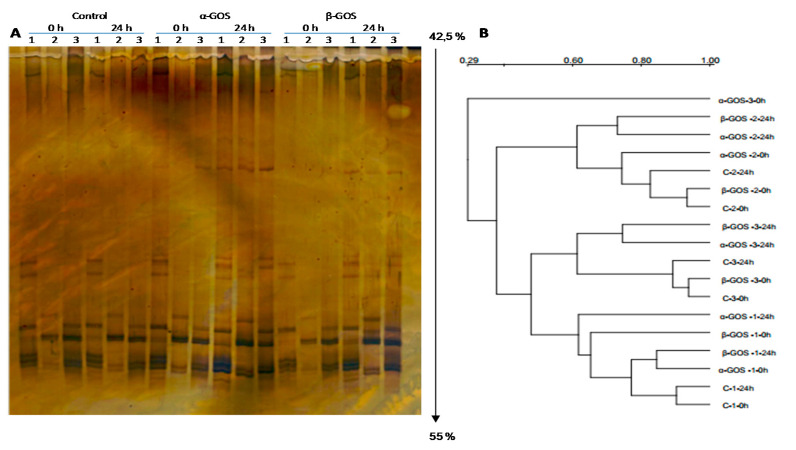

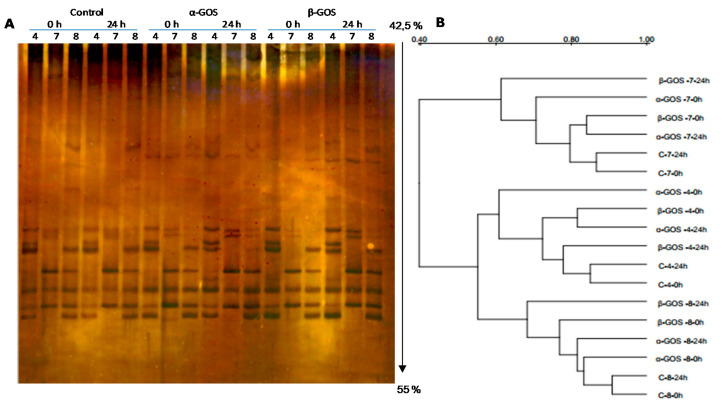
(**A**) DGGE profiles and (**B**) dendrogram of the Bifidobacterium population from faecal samples of six infants and three experimental groups (control, non-fructosylated α-galactooligosaccharides (α-GOS) and β-galactooligosaccharides (β-GOS)) at time 0 and 24 h. Samples were grouped in two gels corresponding to 1, 2, 3 and 4, 7, 8 infants, respectively. Cluster analysis of Denaturing Gradient Gel Electrophoresis (DGGE) pattern profiles was performed using the Dice similarity coefficient and the unweighted-pair group method by means of arithmetic average clustering algorithm (UPGMA). The vertical arrow shows the direction and concentration of the denaturing gradient.

As previously pointed out, non-fructosylated α-GOS and β-GOS had a strong bifidogenic effect in infant inoculum after 6 h of fermentation and was kept at 24 h of treatment. To obtain a more comprehensive assessment of the impact of both GOS on the population structure of bifidobacteria in children’s faecal samples, 16S rRNA gene profiles were generated by means of genus-specific primers PCR-DGGE. Control samples reflected an inter-individual variation in faecal bifidobacterial community being grouped in two cladograms with electrophoretic bands differing from 3 up to 11 (Supplementary Materials Figure S1). Dendrogram analysis indicated a grouping of the samples by children but they were not clustered after non-fructosylated α-GOS and β-GOS treatment (Figure 1). The electrophoretic bands were excised for sequencing and bifidobacterial species identified. Sequencing allowed us to identify *B. catenulatum*/*pseudocatelunatum*, *B. longum* subsp. *longum and B. longum* subsp. *infantis* as the most frequently present bifidobacterial species in infants. Whereas *B. longum* subsp. *infantis* was clearly identified, *B. longum* subsp. *longum* did not achieved such level of recognition at subspecies level and might be an experimental limitation that needs to be considered. The presence of *B. adolescentis and B. bifidum* was identified in only one of the infants. These species have been reported in many studies as the dominant bifidobacteria in infant faecal samples. Significant differences in bifidobacteria communities display differential metabolic features and have strong implications in infant physiology state and health [32,33,34]. Delivery mode (vaginal birth or assisted delivery) and feeding modes (breastfeeding, milk formula and mix-fed) have influences on bifidobacterial composition at species level [32]. Significant changes in the gut microbiota of infants occur when cessation of breastfeeding and introduction of solid foods cause an important shift in gut microbiota composition and begins to resemble a stable adult-like microbiome [35]. Under these circumstances, the supplementation of infant formulae with prebiotic oligosaccharides (GOS/FOS) results in significantly higher stool colony counts of bifidobacterial; it has also been associated to clinical effects such as increased stool frequency and stool softening [36].

To provide an interpretation of the DGGE pattern, three diversity indices of DGGE profiles were calculated (Table 3). The number of bands or richness was significantly higher (*p* < 0.05) in β-GOS after 24 h of fermentation compared to control and non-fructosylated α-GOS that only demonstrated a trend. Shannon and Evenness indices followed the same pattern for each GOS treatment.

### 3.3. Modulation in Bifidobacteria Species Population by Non-Fructosylated α-GOS and β-GOS

In this study, five different bifidobacterial species were quantified at 0 and 24 h after fermentation of non-fructosylated α-GOS and β-GOS with fecal slurries of eight infants (Table 4). The highest level of bifidobacterial species in fecal samples corresponded to *B. longum subsp- longum* and *B. catenulatum/pseudocatenulatum*. It is well-known that bifidobacterial species show differential ability for breakdown, uptake and utilization of carbohydrates. In particular, non-fructosylated α-GOS and β-GOS significantly stimulated the selective growth of *B. longum* subsp. *longum* after 24 h fermentation whereas *B. adolescentis* and *B. longum* subsp. *infantis* were not affected. A significant (*p* < 0.05) growth of *B. catenulatum/pseudocatenulatum* after 24 h fermentation of non-fructosylated α-GOS was observed. Although *B. breve* is a common intestinal bacteria in children’s microbiota, mainly in those fed breast milk, attempts to quantify *B. breve* by qPCR was unsuccessful, likely due to their low levels in fecal samples of children. No match for *B. breve* was found when the faecal bifidobacterial community of children was analyzed by DGGE. In both GOS groups, a significant (*p* < 0.05) decrease in *B. bifidum* population compared to controls was shown. The requirement of prebiotic specificity has recently been questioned by several authors [35,36]. Such controversy is likely to affect to more complex dietary carbohydrates that need bacteria consortium for their degradation instead of GOS compounds that have demonstrated to be selectively bifidogenic [37,38]. Sequencing analysis of fecal samples from healthy human volunteers consuming GOS revealed an increase of bifidobacterial populations [39]. Access to genome sequences of several bifidobacterial strains from infant fecal isolates such as *B. bifidum* PRL2010, *B. breve* UCC2003 and *B. longum* subsp. *infantis* ATCC15697 has increased our knowledge of the metabolic machinery within the genus *Bifidobacterium*. Bifidobacteria contains a high number of glycosyl hydrolases-encoding genes with ability to hydrolyze a wide range of complex carbohydrates (e.g., amylose, amylopectin, maltodextrin) as well as stachyose, raffinose and melibiose, which represent carbohydrates widely present in the human diet [40,41]. Further studies regarding the bifidobacterial enzymes involved in the selective degradation of non-fructosylated α-GOS will allow us to understand their potential as prebiotic ingredient in infant formula.

### 3.4. Lactate and Short-Chain Fatty Acid (SCFA) Concentration

Under anaerobic conditions, non-digestible dietary carbohydrates including GOS are fermented by gut microbiota and thereby producing SCFA that contributes to metabolic regulation and control of energetic metabolism of the host [42]. In our study, lactate and SCFAs were quantified after fermentation of non-fructosylated α-GOS and β-GOS (Table 5). Acetate was the most abundant SCFA, showing a significant (*p* < 0.05) increase after both GOS treatments. In the case of non-fructosylated α-GOS, the levels of acetate increased up to four times compared to baseline. The highest amount of lactate in infant fecal cultures was reported after 6 h in both GOS fermentations, being observed a decrease at 24 h likely due that normally lactate behave as intermediate in microbial metabolism because of onward conversion [43]. Similar to acetate, lactate concentration was higher in non-fructosylated α-GOS compared to β-GOS. Lactate and acetate are main fermentation products of bifidobacteria and are used by other bacteria species to produce butyrate and propionate. In our study, the amounts of propionate and butyrate were not affected after GOS treatment. In agreement with previous studies, GOS are highly selective for *Bifidobacterium* spp. and stimulated acetate production but not the production of propionate and butyrate [44]. Formate amounts were increased due to GOS fermentation, being formate levels after non-fructosylated α-GOS fermentation much higher compared to β-GOS, tripling its value after 24 h (Table 5). Other metabolic indicators such as iso-butyrate, valerate, iso-valerate and succinate were also quantified in this study but no significant changes were observed. The production of SCFA caused a significant drop of pH (0.8 and 1.0 units after 24 h treatment of β-GOS and non-fructosylated α-GOS, respectively) in fecal cultures. Such decrease in luminal pH has been associated to beneficial effects including a decrease in the growth of potential intestinal pathogens sensitive to pH, like some enterobacteria, as well as an increase in the mineral absorption of calcium, magnesium and likely iron [45].

### 3.5. Consumption of Non-Fructosylated α-GOS Per Donor and Degree of Polymerization

**Figure 2:**
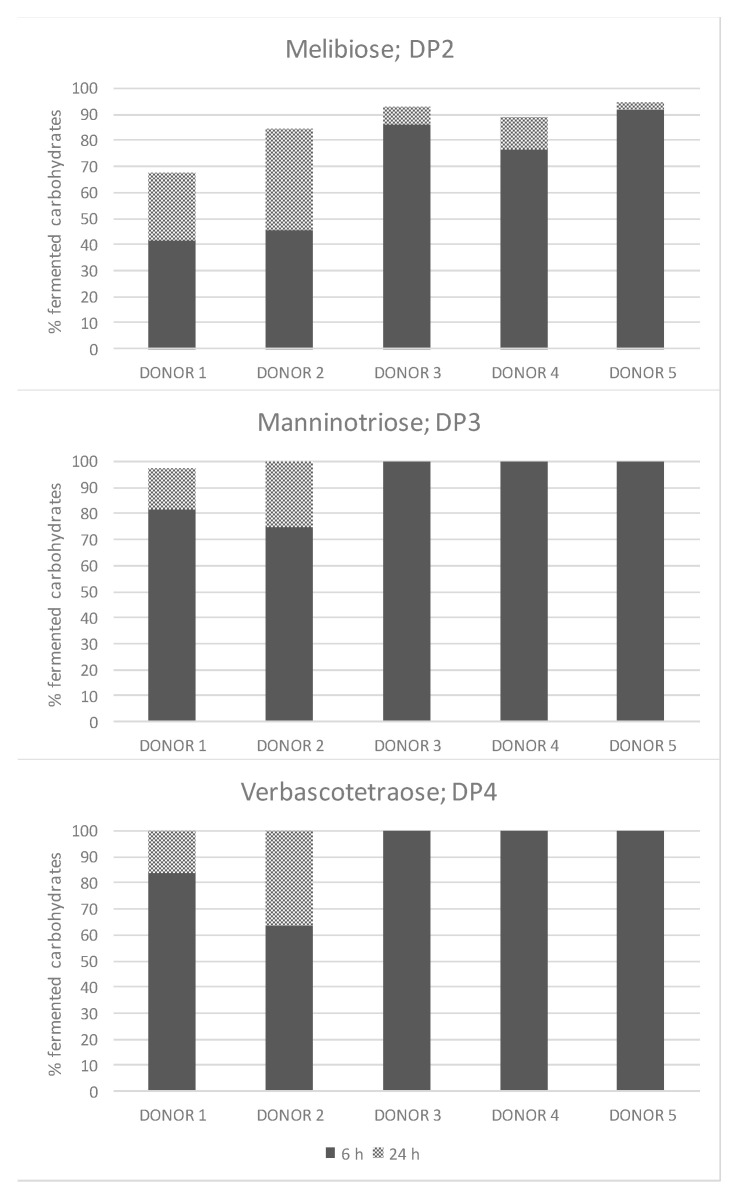
Relative percentage (%) of fermented melibiose (DP2), manninotriose (DP3) and verbascotetraose (DP4) following fermentation of non-fructosylated α-GOS with infant faecal samples (*n* = 5). Darker and lighter colours on the bars indicate the percentage of metabolized carbohydrate after 6 and 24 h of incubation, respectively.

The genome sequences of bifidobacteria display a remarkable enrichment in genes involved in breakdown, uptake, and utilization of a wide variety of resistant carbohydrates to digestion, playing an important role in the healthy infant gut. In this study, manninotriose (DP3) and verbascotetraose (DP4), accounting for more than 92% of total carbohydrates, were completely and readily fermented by the studied donors (Figure 2). This trend was not observed for the disaccharide melibiose, where the fermentation rate ranged between 67% and 95%. Regarding consumption of total non-fructosylated α-GOS, the observed values ranged from 97.6% (donor 1) to 99.9% (donor 5) after 24 h fermentation.

Bifidobacteria can utilize a diverse range of carbohydrates that escape degradation in the upper part of the GIT, as evidenced by the clear enrichment for genes encoding carbohydrate-active enzymes [41]. Bifidobacteria have different specialized oligosaccharide transport systems, since most of their saccharolytic enzymes are intracellular [43]. The fermentation of non-fructosylated α-GOS, as shown in Table 4, significantly increased the growth of *B. longum* subsp. *longum* and *B. catenulatum/pseudocatenulatum* and decreased that of *B. bifidum*. The uptake of α-(1→6)-linked dietary oligosaccharides (ie., raffinose and a mixture of isomaltooligosaccharides) by several *Bifidobacterium* strains, that included *B. longum* subsp. *longum* ICM1217 and *B. longum* subsp. *infantis* ATCC15697 among others, was reported to be mediated by the solute binding protein (B1G16BP) associated with an ATP binding cassette (ABC) transporter [46]. Interestingly, *B. bifidum*, which lacks the genes encoding the B1G16BP ABC transporter, was the only one that did not grow on raffinose and isomaltooligosaccharides, which is in line with our findings. The ABC transporter uses energy produced by the ATP hydrolysis to carry out the oligosaccharides transportation to the intracellular compartment. Once internalized, these oligosaccharides are metabolized by glycosyl hydrolases (GHs) which hydrolyze the glycosidic bond between two or more carbohydrates. Melibiose and raffinose has been reported to be hydrolyzed by *B. longum* subsp. *longum* NCC2705 [40]. According to our results, the lower consumption of melibiose over manninotriose and verbascotetraose could be due to the higher preference of α-galactosides for Gal-(1→6)-Gal-linkages over Gal-(1→6)-Glc, and likely to potential preference for higher DPs. In addition, ligand preference of B1G16BP was ~35 fold lower for melibiose compared with raffinose, which might be explained by its high binding affinity for the non-reducing α-(1→6)-diglycoside, an structural motif present in manninotriose and verbascotetraose but not in melibiose [46]. Although it is clear major metabolic differences might occur at species level, such metabolic abilities might differ considerably between bifidobacterial strains. Thus, it has been reported that β-GOS fermentation is strain-dependent based on their DP and glycosidic linkages [47].

Outcomes were analyzed using a linear mixed model: repeat measure. A Bonferroni method was used for adjustments for major effects (time of treatment and type of GOS). ^a, b^ Mean value with different letters means that differences among treatments was significantly different at *p*-value < 0.05. Unadjusted mean values *n* = 8. Bs: Baseline like covariate. *Interaction among major effects.

## 4. Conclusions

The in vitro fermentation profile of non-fructosylated α-GOS by using infant fecal slurries has been investigated. Remarkable bifidogenic activity of non-fructosylated α-GOS was observed, similar to that obtained using commercial β-GOS, as well as a significant decrease of enterobacteria. A selective and significant increase in *B. longum* subsp. *longum* and *B. catenulatum/psudo-catelunatum* was observed. Therefore, we conclude that these plant-derived GOSs could be used as a prebiotic in infant formula.

## Figures and Tables

**Table 1:** Chemical composition of AlphaGOS^®^.

Parameter	% (Dry Matter)
Dry matter ^a^	96.3
Crude proteins ^a^	<0.2
Crude ash ^a^	<0.2
DP1 + Maltose + Sucrose ^b^	<0.2
DP2: Melibiose ^b^	3.9
DP3: Manninotriose ^b^	49.2
DP4: Verbascotetraose ^b^	43.0

^a^ Provided by the supplier; ^b^ quantified by GC-FID (see Section 2.10 for details); DP: degree of polymerization.

**Table 2:** Microbiota population from in vitro-fermented infant faecal samples with non-fructosylated α-GOS and commercial β-GOS derived from lactose.

	Control			α-GOS			β-GOS			*p*-Values				Pooled SEM
Log10 Copy Number/ Gr Dry Faeces	Bs	6 h	24 h	Bs	6 h	24 h	Bs	6 h	24 h	T	Prebiotics	T * Prebiotics	Bs	
All bacteria	9.78	9.83	9.75	9.77	9.79	9.79	9.89	9.77	9.83	0.871	0.316	0.600	<0.001	0.027
*Bifidobacteria *spp.	7.49	7.59 ^a^	7.68 ^a^	8.01	8.59 ^b^	8.62 ^b^	7.61	8.18 ^b^	8.35 ^b^	0.285	<0.001	0.823	<0.001	0.043
*Clostridium coccoides/* *Eubacterium rectale group*	8.54	8.60	8.54	8.63	8.61	8.66	8.61	8.53	8.55	0.994	0.144	0.606	<0.001	0.020
*Lactobacilli *spp.	5.01	4.78	4.40	5.03	4.86	5.02	5.02	4.69	4.80	0.788	0.150	0.208	<0.001	0.068
*Clostridium leptum subgroup*	7.57	7.67	7.73	7.72	7.68	7.69	8.00	7.93	7.88	0.900	0.049	0.719	<0.001	0.026
*Enterobacteria *spp.	7.81	7.85 ^b^	7.88 ^b^	8.24	8.12 ^a, b^	8.12 ^a, b^	7.93	7.69 ^a^	7.70 ^a^	0817	<0.001	0.967	<0.001	0.028
*F. praustnizii*	8.13	8.14	8.12	8.20	8.09	8.10	8.13	8.04	7.99	0.716	0.100	0.893	<0.001	0.023
Bacteroides	9.63	9.67	9.61	9.67	9.59	9.60	9.63	9.51	9.54	0.855	0.065	0.707	<0,001	0.021

Outcomes were analyzed using a linear mixed model: repeat measure. A Bonferroni method was used for adjustments for major effects (time of treatment and type of GOS) ^a,b^ Mean value with different letters means that differences among treatments was significantly different at *p*-value < 0.05. Unadjusted mean values *n* = 8. Bs: Baseline like covariate. * Interaction among major effects.

**Table 3:** Diversity indices of bifidobacterial samples after non-fructosylated α-GOS and β-GOS 24 h treatment of infant faecal samples.

	Time	Enrichment		Shannon Index		Evenness Index	
		Mean	SD	Mean	SD	Mean	SD
Control	0 h	8.33 ^a^	4.03	2.04 ^a^	0.42	0.65 ^a^	0.13
	24 h	8.33 ^a^	4.03	2.04 ^a^	0.42	0.65 ^a^	0.13
α-GOS	0 h	10.83 ^a^	3.97	2.33 ^a^	0.36	0.74 ^a^	0.11
	24 h	12.50 ^a^	4.68	2.47 ^a^	0.36	0.78 ^a^	0.10
β-GOS	0 h	7.50 ^a^	2.07	1.99 ^a^	0.26	0.63 ^a^	0.08
	24 h	12.00 ^b^	3.16	2.46 ^b^	0.24	0.78 ^b^	0.07

^a, b^, within each row, mean values bearing a different superscript letter differ significantly (*n* = 6; *p* < 0.05). Significance main effects were determined by GLM REP (General Linear Model by one-way ANOVA).

**Table 4:** Effect of non-fructosylated α-GOS and β-GOS on bifidobacterial species of infant faecal samples.

	Control		α-GOS		β-GOS		*p*-Values		Pooled SEM
Log10 Copy Number/Gr De Contenido Fecal	0 h	24 h	0 h	24 h	0 h	24 h	Pb	Bs	
*Bifidobacterium adolescentis*	4.45	4.68	4.65	5.11	4.52	4.52	NS	<0.001	0.193
*Bifidobacterium bifidum*	4.55	5.09 ^b^	4.70	4.04 ^a^	4.57	4.27 ^a^	0.045	<0.001	0.157
*Bifidobacterium catenulatum/pseudo-catenulatum*	7.47	7.41 ^a^	7.25	7.65 ^b^	7.44	7.74 ^a, b^	0.068	<0.001	0.094
*Bifidobacterium longum* subsp. *infantis*	4.96	5.12	5.22	5.40	5.17	5.48	NS	<0.001	0.117
*Bifidobacterium longum* subsp. *longum*	8.25	8.26 ^a^	8.03	8.47 ^b^	8.36	8.71 ^b^	0.010	<0.001	0.065

Outcomes were analyzed using a linear mixed model: repeat measure. A Bonferroni method was used for adjustments for major effects (time of treatment and type of GOS). ^a, b^ Mean value with different letters means the differences among treatments was significantly different at *p*-value < 0.05. Unadjusted mean values *n* = 6. Pb: Prebiotics. Bs: Baseline like covariate.

**Table 5:** Concentration (mM) of lactic acid and short-chain fatty acids (SCFA) infant faecal samples after non-fructosylated α-GOS and β-GOS fermentation.

SCFA (mM)	Control			α-GOS			β-GOS			*p*-Values				Pooled SEM
	Bs	6 h	24 h	Bs	6 h	24 h	Bs	6 h	24 h	T	Prebiotics	T * Prebiotics	Bs	
Acetate	14.669	21.640 ^a^	18.908 ^a^	10.808	44.356 ^b^	45.752 ^b^	16.587	33.463 ^b^	40.743 ^b^	0.671	0.001	0.677	0.084	2.313
Propionate	3.163	6.491	6.116	2.915	9.130	8.522	3.971	8.196	10.072	0.839	0276	0.746	0.060	0.729
Butyrate	3.408	6.664	4.900	6.727	6.970	6.772	7.512	7.719	8.112	0.728	0714	0.831	0.044	0.746
Iso-butyrate	0.307	0.649	0.764	0.253	0.700	0.791	0.339	0.582	0.624	0.540	0.605	0.975	0.412	0.067
Valerate	1.174	4.228	4.788	1.139	2.216	0.607	1.197	2.102	2.249	0.779	0.059	0.678	0.000	0.531
Iso-valerate	0.317	0.635	0.827	0.303	0.775	0.719	0.236	0.602	0.657	0.682	0.897	0.806	0.052	0.077
Formate	0.611	2.400 ^a^	2.447 ^a^	0.523	3.651 ^b^	8.954 ^b^	0.785	4.042 ^a, b^	3.115 ^a, b^	0.258	0.046	0.117	0.390	0.643
Succinate	0.265	1.022	1.022	0.428	1.065	0.975	0.354	1.018	0.969	0.899	0.998	0.995	0.967	0.182
Lactate	0.248	0.782 ^a^	0.500 ^a^	0.211	3.908 ^b^	1.554 ^b^	0.343	1.353 ^a, b^	0.559 ^a, b^	0.028	0.009	0.225	0.015	0.250

Outcomes were analyzed using a linear mixed model: repeat measure. A Bonferroni method was used for adjustments for major effects (time of treatment and type of GOS). ^a, b^ Mean value with different letters means that differences among treatments was significantly different at *p*-value < 0.05. Unadjusted mean values *n* = 8. Bs: Baseline like covariate. * Interaction among major effects.

## Supplementary Materials

The following are available online at https://www.mdpi.com/2304-8158/9/7/921/s1, Figure S1: (A) DGGE profiles and (B) dendogram of the *Bifidobacterium* population from faecal samples of infants, Table S1: β-GOS composition after purification by size-exclusion chromatography, Table S2: PCR primers based on 16S rRNA sequences used for quantitative PCR for bacterial groups, Table S3: PCR primers based on 16S rRNA sequences used for quantitative PCR for Bifidobacteria species.
