# Supplementary material for: Prebiotic Properties of Non-Fructosylated α-Galactooligosaccharides from PEA (Pisum sativum L.) Using Infant Fecal Slurries
Source: Foods. 2020 Jul 13;9(7):921. doi: 10.3390/foods9070921 (PMC7405007; doi:10.3390/foods9070921)
Supplement: Supplementary file 1 [file foods-09-00921-s001.pdf]

Supplemental Figure 1. A) DGGE profiles and B) dendrogram of the Bifidobacterium population from faecal samples from eight infants

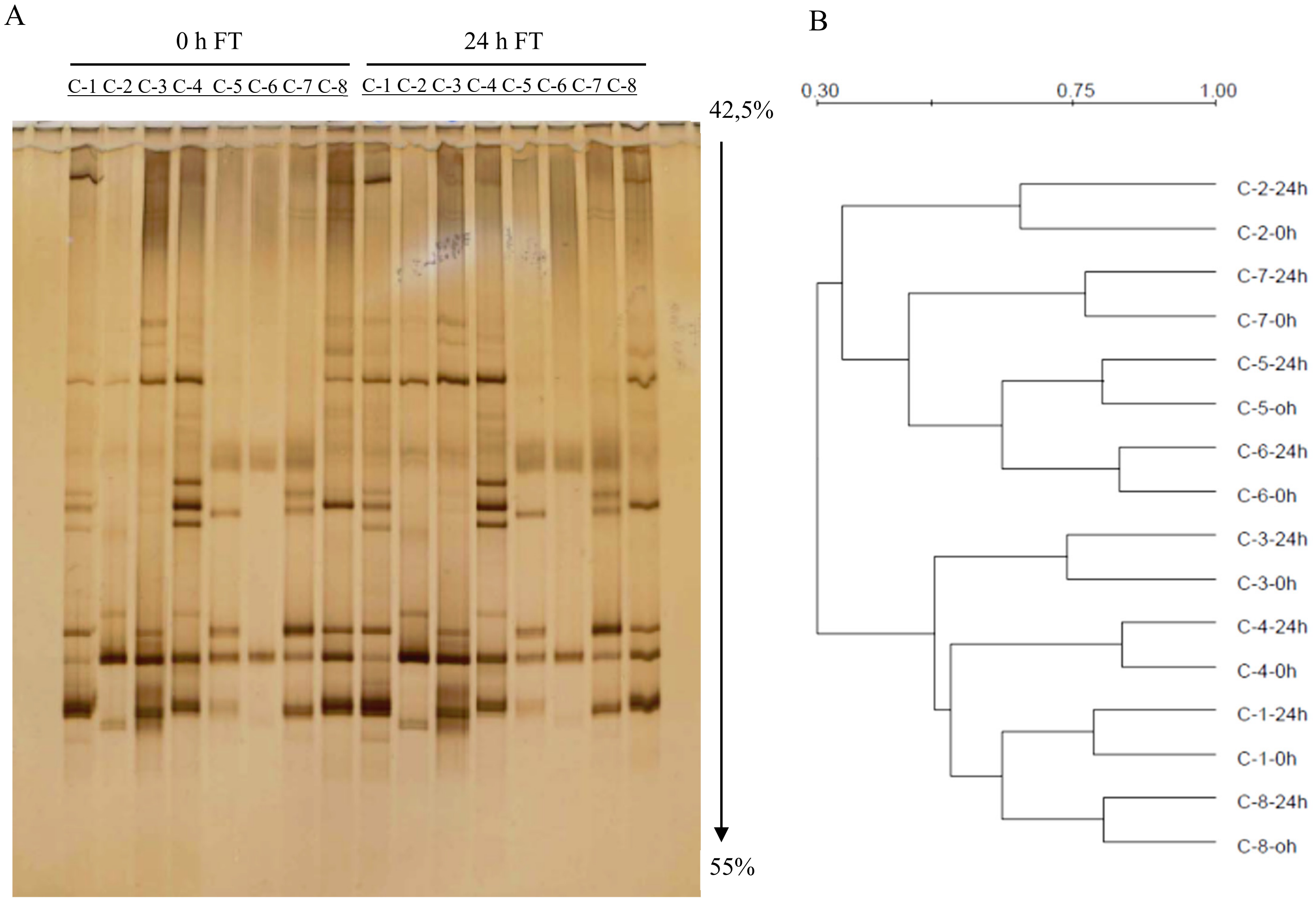

Faecal control samples from infants at 0 and 24 h of fermentation. Cluster analysis of DGGE pattern profiles was performed using the Dice similarity coefficient and the unweighted-pair group method by means of arithmetic average clustering algorithm (UPGMA). Vertical arrow shows the direction and concentration of the denaturing gradient.

Supplemental Table 1. β-GOS composition after purification by size-exclusion chromatography (SEC)

**β-GOS purified by SEC**	
**Structures**	**% (w/w)**
β-Gal-(1-4)-β-Gal-(1-4)-Glc	15.8
β-Gal-(1-6)-β-Gal-(1-4)-Glc	5.3
β-Gal-(1-4)-β-Gal-(1-6)-Glc	3.2
β-Gal-(1-4)-β-Gal-(1-2)-Glc	3.2
β-Gal-(1-6)-β-Gal-(1-2)-Glc	1.8
Unknown DP3	5.9
DP4 + DP5 + DP6	64.8

Supplemental Table 2. PCR primers based on 16S rRNA sequences used for quantitative PCR for bacterial groups.

Target bacterial group	*Primer*	*Oligonucleotide Sequence (5'–3')*	*PCR product size (bp)*	*Annealing Temp (°C)*	*References*
All bacteria	F-Eub 338<br>R-Eub 518	ACTCCTACGGGAGGCAGCAG<br>ATTACCGCGGCTGCTGG	200	60	Guo *et al*. (2008)
Bacteroides	F-AllBac 296<br>R-AllBac 412	GAGAGGAAGGTCCCCCAC<br>CGCTACTTGGCTGGTTCAG	106	60	Layton *et al*. (2006)
Bifidobacteria	F-Bifido<br>R-Bifido	CGCGTCYGGTGTGAAAG<br>CCCCACATCCAGCATCCA	244	60	Delroisse *et al.* (2008)
Clostridium coccoides / Eubacterium rectale group	F-g-Ccoc<br>R-g-Ccoc	AAATGACGGTACCTGACTAA<br>CTTTGAGTTTCATTCTTGCGAA	440	50	Matsuki *et al*. (2004a)
Clostridium leptum subgroup	F-sg-Clept<br>R3-sg-Clept	GCACAAGCAGTGGAGT<br>CTTCCTCCGTTTTGTCAA	239	50	Matsuki *et al*. (2004a)
Lactobacilli	F-Lacto<br>R-Lacto	GAGGCAGCAGTAGGGAATCTTC<br>GGCCAGTTACTACCTCTATCCTTCTTC	126	60	Delroisse *et al.* (2008)
Enterobacteria	F-Ent<br>R-Ent	ATGGCTGTCGTCAGCTCGT<br>CCTACTTCTTTTGCAACCCACTC	385	60	Castillo *et al*. (2006)

Supplemental Table 3. PCR primers based on 16S rRNA sequences used for quantitative PCR for Bifidobacteria species.

Target bacterial group	*Primer*	*Oligonucleotide Sequence (5'–3')*	*PCR product size (bp)*	*Annealing Temp (°C)*	*References*
Bifidobacterium adolescentis	BiADO-1a<br>BiADO-2	CTCCAGTTGGATGCATGTC<br>CGAAGGCTTGCTCCCAGT	279	55	Matsuki *et al.* (2004b)
Bifidobacterium bifidum	BiBIF-1<br>BiBIF-2	CCACATGATCGCATGTGATTG<br>CCGAAGGCTTGCTCCCAAA	278	55	Matsuki *et al.* (2004b)
Bifidobacterium catenulatum/pseudo-catenulatum	BiCATg-1<br>BiCATg-2	CGGATGCTCCGACTCCT<br>CGAAGGCTTGCTCCCGAT	285	55	Matsuki *et al.* (2004b)
Bifidobacterium infantis	BiINF-1<br>BiINF-2	TTCCAGTTGATCGCATGGTC<br>GGAAACCCCATCTCTGGGAT	828	55	Matsuki *et al.* (2004b)
Bifidobacterium longum	BiLON-1<br>BiLON-2	TTCCAGTTGATCGCATGGTC<br>GGGAAGCCGTATCTCTACGA	829	55	Matsuki *et al.* (2004b)
Bifidobacterium breve	BiBRE-1<br>BiBRE-2	CCGGATGCTCCATCACAC<br>ACAAAGTGCCTTGCTCCCT	288	55	Matsuki *et al.* (2004b)
Faecalis prausnitzii	JFF-F-praust-up<br>JFF-F-praust-rp	TTAACACAATAAGTAATCCACCTGG<br>ACCTTCCTCCGTTTTGTCAAC	314	60	

References.

Delroisse, J.M., Bolvin, A.L., Parmentier, I., Dauphin, R.D., Vandenbol, M., Portetelle, D., 2008. Quantification of Bifidobacterium spp. and Lactobacillus spp. in rat fecal samples by real-time PCR. Microbiol. Res. 163, 663-670.

Guo, X., Xia, X., Tang, R., Zhou, J., Zhao, H., Wang, K., 2008. Development of a real-time PCR method for Firmicutes and Bacteroidetes in faeces and its application to quantify intestinal population of obese and lean pigs. Lett. Appl. Microbiol. 47, 367-373.

Layton, A., McKay, L., Williams, D., Garrett, V., Gentry, R., Sayler, G., 2006. Development of Bacteroides 16S rRNA gene Taqman-based real-time PCR assays for estimation of total, human and bovine fecal pollution in water. Appl. Environ. Microbiol. 72, 4214-4224.

Marisol Castillo, Susana M. Martín-Orúe, Edgar G. Manzanilla, Ignacio Badiola, Marga Martín, Josep Gasa. 2005. Quantification of total bacteria, enterobacteria and lactobacilli populations in pig digesta by real-time PCR. Veterinary Microbiology.

Takahiro Matsuki, Koichi Watanabe, Junji Fujimoto, Toshihiko Takada, and Ryuichiro Tanaka. 2004a. Use of 16S rRNA Gene-Targeted Group-Specific Primers for Real-Time PCR Analysis of Predominant Bacteria in Human Feces. Appl. Environm. Microbiol, p. 7220–7228.

Takahiro Matsuki, Koichi Watanabe, Junji Fujimoto, Yukiko Kado, Toshihiko Takada, Kazumasa Matsumoto, and Ryuichiro Tanaka. 2004b. Quantitative PCR with 16S rRNA-Gene-Targeted Species-Specific Primers for Analysis of Human Intestinal Bifidobacteria. Appl. Environm. Microbiol, p. 167–173.
